# Characteristics and outcomes of a hospitalized cohort with reduced mortality from COVID-19, White Mountain apache tribal lands, April 1 – July 31, 2020

**DOI:** 10.1186/s12889-024-18098-5

**Published:** 2024-03-01

**Authors:** Ryan M. Close, Chelsea S. Lutz, T. Shaifer Jones, Myles Stone, Nicole Bratsch, Trevor Thompson, Christopher Jentoft, James B. McAuley

**Affiliations:** 1 Whiteriver Service Unit, Indian Health Service, 200 W. Hospital Drive, Whiteriver, AZ 85941 USA; 2 Maine Medical Center, MaineHealth, Portland, ME USA; 3 Department of International Health, Johns Hopkins University Bloomberg School of Public Health, Baltimore, MD USA; 4 United States Public Health Service Commissioned Corps, Rockville, MD USA

**Keywords:** American Indian, COVID-19, Hospitalizations, Outreach

## Abstract

**Background:**

Widespread transmission of COVID-19 continues to threaten public health, particularly of rural, American Indian communities. Although COVID-19 risk factors for severe disease and clinical characteristics are well described in the general population, there has been little shared on hospitalized American Indian populations.

**Methods:**

In this observational study, we performed chart extractions on all persons hospitalized with COVID-19 from April 1 through July 31, 2020 among an exclusively American Indian population living on or near Tribal lands in eastern Arizona. We provide descriptive statistics for the cohort stratified by presentation, comparing those who self-presented or were referred by an outreach program. Exploratory analyses were performed to identify risk factors for morbidity and mortality.

**Results:**

During the observation period, 2262 persons were diagnosed with COVID-19 and 490 (22%) were hospitalized. Hospitalized persons had a median age of 54 years; 92% had at least one comorbidity, 72% had greater than one comorbidity, and 60% had a BMI of > 30. Most persons required supplemental oxygen (83%), but the majority (62%) only required nasal cannula and only 11% were intubated. The case fatality rates were 1.7% for the population, 7.1% among hospitalizations, and 9.3% among hospitalized patients 50 years and older. All rates that are significantly lower than those reported nationally during the same period.

**Conclusions:**

We observed a cohort of American Indian patients hospitalized secondary to COVID-19 with greater number of comorbidities compared to the general population but with lower mortality rates. We posit that the primary driver of mortality reduction for this population and the hospitalized cohort was a community-based referral program that led to disproportionately lower fatality rates among the oldest persons.

**Supplementary Information:**

The online version contains supplementary material available at 10.1186/s12889-024-18098-5.

## Background

SARS-CoV-2, the virus responsible for coronavirus disease 2019 (COVID-19), continues to affect populations across the United States (US) and remains a threat to public health. American Indian (AI) communities have been disproportionately impacted by the pandemic. By June 2020, American Indian populations in the US were experiencing incidence and mortality rates 3.5 and 1.8 times greater than White Americans, respectively [1, 2]. The clinical characteristics of COVID-19 are well documented in the general population [3–10], but there is little research on American Indian populations. Available studies aggregate data to establish general population-level trends, but fail to draw conclusions regarding the effects of specific underlying conditions [1, 2]. Even studies that have focused on racial health disparities have limited data from American Indian communities [11–14].

What data is available suggests that American Indian communities have experienced significantly greater excess COVID-19 and non-COVID-19 mortality since the start of the pandemic. However, there are critical knowledge gaps regarding the epidemiology of severe COVID-19 and disease progression leading to hospitalization among American Indian persons. To address this need, we present descriptive data of COVID-19 hospitalizations occurring April 1 through July 31, 2020 among American Indian persons residing on or near the White Mountain Apache Tribal lands. We explore associations between medical outcomes and the effects of a high-risk home outreach referral program that formed an integral part of the local pandemic response.

## Methods

### Setting and population

The Fort Apache Indian Reservation spans over 2600 mile [2] in eastern Arizona and is home to an American Indian population of approximately 18,000, (pop. Density 7 persons per square mile) that are predominantly members of the White Mountain Apache Tribe. The Whiteriver Service Unit (WRSU) includes an acute care hospital that serves as the primary health care center and public health authority for the local community. The hospital consists of a 40-bed inpatient unit, several outpatient clinics, as well as a pharmacy and emergency department that are open 24-hours a day. There is a satellite clinic 1 hour away. The majority of the population lives within 25 miles of either the satellite clinic or the main hospital. The nearest tertiary care center is 4 hours away by ground transport. The WRSU does not have an intensive care unit (ICU) and all patients requiring invasive support and monitoring are transferred to higher levels of care. Charts eligible for review included all persons registered at the WRSU who reside on or near the Fort Apache Indian Reservation with a positive test for SARS-CoV-2 via molecular testing and hospitalized for COVID-19 related illness April 1 through July 31, 2020.

### Structure of home-visit program

During the pandemic, WRSU used a high-risk home-visit outreach referral program to monitor persons at high-risk for severe COVID-19, including hospitalization, and tracked all of those referred for early intervention. The program has been outlined in detail previously by the authors [15]. In summary, the WRSU reviewed the charts of all patients who tested positive for SARS-CoV-2 via molecular testing and identified those with increased likelihood of severe COVID-19, this included but was not limited to: Age ≥ 60 years, body mass index (BMI) ≥ 30, medical history of diabetes mellitus, chronic kidney disease, chronic lung disease, end stage liver disease, or immunosuppressive disease or treatment. Patients with the applicable criteria received in-person daily home visits from a WRSU paraprofessional who monitored subjective symptoms and vital signs, including oxygen saturation at rest and with ambulation. Patients were monitored until they displayed notable improvement and safe for discharge from the program or were referred to the WRSU emergency department for further evaluation.

In parallel to the high-risk home visit program, an innovative case investigation and contact tracing (CI/CT) program operated that has been detailed by authors previously [16]. CI/CT was performed largely in-person via home visit as well, offering additional field testing to household members of cases, particularly those household contacts with high-risk criteria listed above.

### Data collection

Charts of hospitalized eligible persons were reviewed to extract demographic information (age, sex), medical history, SARS-CoV2 testing, and comorbidities. Information was collected by “presentation mode”: whether persons were referred by field outreach teams or self-presented for care. Additional data was collected on symptom characteristics and onset prior to hospitalization, presenting vital statistics and laboratory test results, and treatments received while hospitalized. Finally, data was collected on medical outcomes, including hospital admission and discharge date, transfer to higher levels of care, oxygen requirements, ICU requirement, and death, regardless of location of hospitalization.

### Statistics

Descriptive statistics were reported using parametric and non-parametric methods where appropriate. Comparative statistics were conducted to explore differences in outcomes based on referral patterns (e.g. team-referred versus self-presentation). To determine which comorbidities were associated with an increased risk of death from COVID-19 within 28 days of hospitalization, we constructed a multiple logistic regression model using backward variable selection and a threshold *p*-value of 0.05. Additionally, we constructed a multiple ordered logistic regression model to determine which demographic factors were associated with increased peak oxygen requirements. No power analysis was conducted given the retrospective, observational nature of this study. All data were analyzed using Stata version 16.1 (StataCorp. 2019. Stata Statistical Software: Release 16. College Station, TX: StataCorp LLC).

### Ethics

The study procedures and publication were approved by the White Mountain Apache Tribal Health Advisory Board and Tribal Council as well as the Institutional Review Board of Phoenix Area Indian Health Service.

## Results

From April 1 through July 31, 2020, 2262 persons tested positive for SARS-CoV-2 via molecular testing at the WRSU, of whom 490 (21.7%) were hospitalized. The demographic characteristics of hospitalized persons are shown in Table 1. Of the 490 persons hospitalized for COVID-19, median age was 54 years (IQR 41–64) and 269 (54.9%) were female. The median body mass index (BMI) was 31.4 (IQR 26.6–37.7), with 60% having a BMI of 30 or greater and nearly 35% having a BMI of 35 or greater. Of all hospitalized persons, 451 (92.0%) had at least one pre-existing condition (Table 1). Almost half (46.7%) of admitted cases had a diagnosis of diabetes and 53.1% had a history of hypertension. Alcohol use disorder (32.2%) and substance abuse (9.6%) were common, especially among younger persons. Among persons under 40 years old, 56% had a history of alcohol abuse and 27% had a history of polysubstance abuse, compared to persons 40 or older, of whom 22% had a history of alcohol abuse and 4% had a history of polysubstance abuse.

**Table 1:** Demographics and co-morbidities of patients hospitalized with COVID-19, total and stratified by presentation

Characteristic	All hospitalized *n* = 490	Self-presenting *n* = 321	Team-referred *n* = 169	*p*-value
Age – years				
Median (IQR)	54 (41–64)	51 (37–61)	58 (47–67)	< .001
Range	0–93	0–89	21–93	
Sex				
Female – no. (%)	269 (54.9)	166 (51.7)	103 (61.0)	.05
Male – no. (%)	221 (45.1)	154 (48.3)	66 (39.1)	
Preexisting conditions				
At least 1 – no. (%)	451 (92.0)	297 (92.5)	154 (91.1)	.59
Body mass index	*n* = 486	*n* = 317	*n* = 169	
Median (IQR)	31.5 (26.6–37.7)	31.7 (27.0–37.9)	31.0 (26.3–36.0)	.33
Range	16.7–75.9	16.7–75.9	18.3–68.3	
≥ 30 – no. (%)	290/486 (59.7)	191/317 (60.3)	99/169 (58.6)	.72
≥ 35 – no. (%)	167/486 (34.4)	114/317 (36.0)	53/169 (31.4)	.31
History of pneumonia – no. (%)	110 (22.5)	74 (23.1)	36 (21.3)	.66
Diabetes – no. (%)	229 (46.7)	140 (43.6)	89 (52.7)	.06
A1C^a^	*n* = 202	*n* = 121	*n* = 81	
A1C – median (IQR)	8.1 (6.8–10.4)	7.9 (6.8–10.4)	8.3 (7.0–10.3)	.49
≥ 7 no./n (%)	151/212 (71.2)	86/217 (67.7)	65/85 (76.5)	.17
Hypertension – no. (%)	260 (53.1)	157 (48.9)	103 (61.0)	.01
Asthma – no. (%)	63 (12.9)	42 (13.1)	21 (12.4)	.84
Chronic obstructive pulmonary disease – no. (%)	25 (5.1)	18 (5.6)	7 (4.1)	.48
Chronic liver disease– no. (%)	57 (11.6)	41 (12.8)	16 (9.5)	.28
Neurological disease – no. (%)	33 (6.7)	21 (6.5)	12 (7.1)	.82
Blood disorder – no. (%)	21 (4.3)	17 (5.3)	4 (2.4)	.13
Coronary artery disease – no. (%)	24 (4.9)	16 (5.0)	8 (4.7)	.90
Congestive heart failure – no. (%)	30 (6.1)	19 (5.9)	11 (6.5)	.80
Chronic kidney disease – no. (%)	54 (11.0)	28 (8.7)	26 (15.4)	.03
Hemodialysis dependent – no. (%)	19 (3.9)	14 (4.4)	5 (3.0)	.27
Cancer (active diagnosis) – no. (%)	14 (2.9)	7 (2.2)	7 (4.1)	.22
Alcohol use disorder – no. (%)	158 (32.2)	117 (36.5)	41 (24.3)	.006
Substance abuse (not-alcohol) – no. (%)	47 (9.6)	37 (11.5)	10 (5.9)	.05
Tobacco use				
Never used – no. (%)	325 (66.3)	211 (65.7)	114 (67.5)	.70
Former User – no. (%)	125 (25.5)	83 (25.9)	42 (24.9)	
Current User – no. (%)	40 (8.2)	27 (8.4)	13 (7.7)	

*A1C* Hemoglobin A1C test, *IQR* interquartile range, *No* number

^a^If A1c collected within 2 years of hospitalization

Overall, symptoms were common prior to hospitalization, with 93.4% reporting at least one symptom (Supplementary Table 1). The most common symptoms at the time of presentation among all hospitalized persons were cough (76.1%), shortness of breath (73.7%), subjective fever (57.9%), and aches (48.7%).

Compared to persons who self-presented, referred persons were older (58 years vs. 51 years), more likely to be female, and more likely to have hypertension and chronic kidney disease (Table 1). Self-presenting persons who were hospitalized for COVID-19 related illness were more likely to have a history of alcohol and polysubstance use, both of which were more common in younger persons (mean age among persons with substance use disorders 44 years, SD 14 years). Symptoms also varied by presentation mode. Referred persons were more likely to have classic viral respiratory symptoms including chills, aches, fatigue, cough, dyspnea, and chest pain whereas self-presenting persons were more likely to have gastrointestinal complaints (Supplementary Table 1).

Presenting vital signs and laboratory values are summarized in Table 2. A documented elevated temperature was uncommon upon presentation, with only 22.9% with a temperature of 38.0 degrees Celsius or higher at presentation. Nearly 80% of all persons had hypoxia, either at rest (65.4%) or with ambulation (14.5%). Self-presenting persons were more likely to be tachycardic (> 100 bpm) or febrile compared to referred persons, and referred persons were more likely to have low oxygen saturation, either at rest or with ambulation (Table 2).

The majority (80.3%) of hospitalized persons had abnormalities on radiography consistent with pneumonia, which was more common among team-referred hospitalized persons (85.7% compared to 77.4% for self-presenting persons).

Lymphopenia was common. The median lymphocyte count was 1149 k/cmm and over a third of hospitalized persons had absolute lymphopenia (count < 1000 k/cmm). Elevations in inflammatory markers like C-reactive protein (CRP) were also common with over a third (34.1%) of hospitalized COVID-19 cases with a CRP > 100 mg/L. An abnormal troponin was detected in 64 (16.2%) of hospitalizations and of those with an abnormal troponin, median detectable value was 0.07 ng/mL (reference range 0–0.04 ng/L). When comparing persons by presentation mode, referred persons were more likely to have lower glomerular filtration rate (GFR) and higher brain natriuretic peptide (BNP) on presentation, whereas self-presenting persons were more likely to have elevated transaminases (aspartate aminotransferase and alanine aminotransferase [AST/ALT]), bilirubin, and creatine phosphokinase (CPK). While statistically significant, it is unclear if these differences are substantive, and they likely reflect differences in demographics and the clinical picture described above.

Admitted patients were commonly treated with antibiotics, with over two-thirds (69%) receiving antibiotics for pneumonia. A third of all persons received steroids and nearly half (48.2%) received remdesivir (Table 3). Compared to self-presenting persons, referred persons were more likely to receive at least one dose of remdesivir (55.8% versus 44.3%) and be treated with an anti-platelet (61.5% versus 51.0%).

**Table 3:** Treatments administered to patients hospitalized with COVID-19, total and stratified by presentation

Medication	All hospitalized *n* = 456	Self-presenting *n* = 294	Team-referred *n* = 162	*p*-value
Antibiotics – no. (%)	314 (69.2)	199 (66.8)	115 (73.7)	.13
Corticosteroids – no. (%)	156 (34.4)	97 (32.6)	59 (37.8)	.26
Remdesivir (any) – no. (%)	219 (48.2)	132 (44.3)	87 (55.8)	.02
Complete course – no./n (%)	146/213 (68.5)	88/127 (69.3)	58/86 (67.4)	.78
Convalescent plasma – no. (%)	53 (11.7)	30 (10.1)	23 (14.7)	.14
Tocilizumab – no. (%)	7 (1.5)	2 (0.7)	5 (3.2)	.04
Venous thromboembolism prophylaxis – no. (%)	416 (91.6)	269 (90.3)	147 (94.2)	.15
Inpatient antiplatelet – no. (%)	248 (54.6)	152 (51.0)	96 (61.5)	.03
Discharge medications				
Discharged with antiplatelet – no. (%)	274/429 (63.9)	170/285 (59.7)	104/144 (72.2)	.01
Discharged with supplementary oxygen – no. (%)	186/425 (43.8)	112/281 (39.9)	74/144 (51.4)	.02

*No* number

Only includes patients with complete treatment information

### Outcomes

Roughly half of all hospitalized persons (46.1%) were admitted and treated locally at the WRSU, while 42.2% of persons were immediately transferred to a higher level of care, and 10.2% were transferred following local admission (Table 4). There were no differences in admission or transfer patterns based on mode of presentation. That is, at the point in which outreach team referrals were complete and medical decision making was the purview of providers in the Emergency Department, there were no differences in the proportion of persons who were admitted locally or transferred to a higher level of care based on presentation mode.

**Table 4:** Outcomes of patients hospitalized with COVID-19, total and stratified by presentation

Characteristic	All hospitalized *n* = 490	Self-presenting *n* = 321	Team-referred *n* = 169	*p*-value
Hospitalization type				
Local admit only	226 (46.1)	144 (44.9)	82 (48.5)	.72
Local admit to transfer	50 (10.2)	31 (9.7)	19 (11.2)	
Immediate transfer	207 (42.2)	141 (43.9)	66 (39.1)	
Direct admit to outside hospital	7 (1.4)	5 (1.6)	2 (1.2)	
COVID-19 death within 28-days	35 (7.1)	17 (5.3)	18 (10.7)	.03
COVID-19 Death (any time)	39 (8.0)	19 (5.9)	20 (11.8)	.02
Days to COVID-19 death				
Median (IQR)	15.0 (10–21)	14.0 (8–21)	15.5 (11.5–22)	.13
Age group				
< 55	8/255 (3.1)	5/188 (2.7)	3/67 (4.5)	.46
55–64	10/122 (8.2)	7/70 (10.0)	3/52 (5.8)	.40
65–74	10/69 (14.5)	4/40 (10.0)	6/29 (20.7)	.30
> =75	11/44 (25.0)	3/23 (13.0)	8/21 (38.1)	.08
ICU	95/463 (20.5)	61/305 (20.0)	34/158 (21.5)	.70
Days in ICU	*n* = 84	*n* = 53	*n* = 31	
Days in ICU – median (IQR)	6 (2.5–15)	6 (2–11)	8 (3–21)	.05
Days to ICU admission	*n* = 89	*n* = 56	*n* = 33	
Median (IQR)	2 (0–4)	1 (0–4)	3 (1–4)	.13
Supplementary oxygen support (any)	387/465 (83.2)	246/305 (80.7)	141/160 (88.1)	Χ^2^ *p* = 0.041
Peak oxygen support	*n* = 465	*n* = 305	*n* = 160	
None	78/465 (16.8)	59/305 (19.3)	19/160 (11.9)	.09
Nasal cannula	289/465 (62.2)	184/305 (60.3)	105/160 (65.6)	
Non-rebreather	14/465 (3.0)	10/305 (3.3)	4/160 (2.5)	
High-flow nasal cannula	17/465 (3.7)	11/305 (3.6)	6/160 (3.8)	
Continuous positive airway pressure support	5/465 (1.1)	5/305 (1.6)	0/160 (0.0)	
Bilevel positive airway pressure support	11/465 (2.4)	6/305 (2.0)	5/160 (3.1)	
Mechanical ventilation (MV)	51/465 (11.0)	30/305 (9.8)	21/160 (13.1)	
MV – available data	*n* = 46	*n* = 26	*n* = 20	
Days requiring MV – median (IQR)	12 (7–22)	11.5 (7–16)	14.5 (8–30)	.27
Interval from admission to MV – n, median, (IQR)	*n* = 46, 4 (1–8)	*n* = 26, 3 (1–8)	*n* = 20, 5 (3.5–7)	0.18

*ICU* intensive care unit, *IQR* interquartile range, *MV* mechanical ventilation, *No* number

Among our cohort of 490 hospitalized persons, there were 39 deaths (8.0%) attributable to COVID-19 during the observation period, 35 (7.1%) of which occurred within 28 days of hospitalization. Median interval from hospitalization to death was 15 days (IQR 10–21 days). Referred persons were twice as likely to die from COVID-19 within 28 days or at any time, compared to self-presenting persons (11.8% vs. 5.9%, respectively). As age increased, the proportion of hospitalized patients that died from COVID-19 also increased. This was true for the whole cohort and when stratified by presentation mode (Table 4).

One-fifth (20.5%) of hospitalized patients ended up in the ICU with a median ICU stay of 6 days (IQR 3–15), and the median interval from hospitalization to ICU admission was 2 days (IQR 0–4) (Table 4). Most persons (83.2%) required some level of supplemental oxygen during hospitalization, but the majority (62.2%) required only nasal cannula with median flow of 2 lpm. Only 11.0% of persons were intubated. There were no significant differences in ICU stays or oxygen requirements by presentation mode. Additional outcomes are summarized in Supplementary Table 2.

Exploratory analyses using multiple logistic regression found that age, male sex, and BMI were associated with COVID-19 related death (overall model pseudo r-squared = 0.16, chi-square = 43.4, *p* < .001, Supplementary Table 3). Additionally, when controlling for these three variables, lower oxygen saturation at time of presentation was also associated with COVID-19 death within 30 days (t = − 4.7, CI − 0.2 to − 0.1, *p* < 0.001). Increasing age and BMI were each associated with increasing oxygen and respiratory support, independent of mortality (overall model pseudo r-squared = 0.12, chi-square = 50.1, *p* < .001, Supplementary Table 4).

## Discussion

Over a four-month period, from April 1 through July 31, 2020, the Whiteriver Service Unit diagnosed 2262 persons with COVID-19, of which 490 (21.7%) were hospitalized and 39 (1.7%) died. Observed deaths included all death within the cohort regardless of location of care, including those requiring ICU admission at outside facilities. This community-wide case fatality rate of 1.7% is significantly lower than what has been reported contemporaneously nationally, statewide in Arizona, and compared to other regional Tribal territories [2, 17, 18].

The observed in-hospital mortality rate of 7.1% was nearly a third of previous reports, which found rates ranging from 21 to 28% (Table 5) [6, 10, 20–22]. While a review by Wiersinga et al. found a slightly lower in-hospital mortality range of 15–20% [23], this lower bound is still double the WRSU observed rate. If we assume all 35 in-hospital deaths were among the 387 (83%) with an oxygen requirement, this provides potentially a more generalizable CFR (9.0%, 35/387) to compare with other settings. This more conservative CFR is still substantially lower than what has been described elsewhere, including the lower and more generalizable weekly in-hospital mortalities reported by the CDC with CFR’s ranging from 8.8 to 19.9%, with only a single week where the mortality rate dropped below 9.0% and half the weeks > 12.0% [24].

**Table 5:** Comparing the Whiteriver Service Unit cohort of patients hospitalized with COVID-19 to published experience

Characteristics	Whiteriver Service Unit	Richardson, et al. [1]	Petrilli, et al. [2]	Zhou, et al. [3]	Reilev, et al. [4]	Docherty, et al. [5]	Garg, et al. [6]
Location	Whiteriver, AZ, USA	New York City, NY, USA	New York City, NY, USA	Wuhan, China	Denmark	United Kingdom	COVID-NET^a^
n	490	5700	2741	191	2254	20,133	18,508
Demographics	n = 490	*n* = 5700	*n* = 2729	*n* = 191	*n* = 2254	n = 20,133	*n* = 18,508
Age – median (IQR)	54 (41–64)	63 (52–75)	63 (51–74)	56 (46–67)	71 (56–80)	73 (58–81)	62 (48–74)
Female sex – no. (%)	269 (55)	2263 [16]	1069 [19]	72 (38)	1042 (46)	8065 (40)	9098 (49)
Obesity – no./n (%)	290/486 (60)	1737/4170 (42)	1081 [19]	–	277 (12)	1685 [11]	–
No. Comorbidities							
At least one – no. (%)	451 (92)	5350 (94)	2176 (71)	91 (48)	1752 (78)	14,364 (78)	14,931 (84)
More than one – no. (%)	351 (72)	4991 (88)	–	–	1268 (56)	–	10,239 (62)
Outcomes							
ICU – no./n (%)	95/463 (21)	373/2634 [14]	749 (27)	50 (26)	314 (14)	3001 (17)	4966 (27)
Intubation – no./n (%)	51/464 (11)	320/2634 [12]	647 (24)	32 (17)	–	1658 [10]	2668 [14]
Death – no./n (%)	35/490 (7)	553/2634 [20]	665 (24)	54 (28)	577 (26)	5165 (26)	1801 [10]

1. Richardson S, Hirsch JS, Narasimhan M, et al. Presenting Characteristics, Comorbidities, and Outcomes Among 5700 Patients Hospitalized With COVID-19 in the New York City Area. JAMA. 2020;323 [21]:2052–2059. Doi:10.1001/jama.2020.6775

2. Petrilli CM, Jones SA, Yang J, et al. Factors associated with hospital admission and critical illness among 5279 people with coronavirus disease 2019 in New York City: prospective cohort study. BMJ. 2020;369:m1966. Doi:10.1136/bmj.m1966

3. Zhou F, Yu T, Du R, et al. Clinical course and risk factors for mortality of adult inpatients with COVID-19 in Wuhan, China: a retrospective cohort study. The Lancet. 2020;395(10229):1054–1062. Doi:10.1016/S0140-6736(20)30566-3

4. Reilev M, Kristensen KB, Pottegård A, et al. Characteristics and predictors of hospitalization and death in the first 11,122 cases with a positive RT-PCR test for SARS-CoV-2 in Denmark: a nationwide cohort. Int J Epidemiol. 2020;49 [5]:1468–1481. Doi:10.1093/ije/dyaa140

5. Docherty AB, Harrison EM, Green CA, et al. Features of 20,133 UK patients in hospital with covid-19 using the ISARIC WHO Clinical Characterisation Protocol: prospective observational cohort study. BMJ. 2020;369:m1985. Doi:10.1136/bmj.m1985

6. Shikha Garg, Kadam Patel, Huong Pham, et al. Clinical Trends Among U.S. Adults Hospitalized With COVID-19, March to December 2020: A Cross-Sectional Study. Ann Intern Med.2021;174:1409–1419. [Epub ahead of print 10 August 2021]. Doi:10.7326/M21-1991

^a^ Coronavirus Disease 2019-Associated Hospitalization Surveillance Network (COVID-NET) is a CDC population-based surveillance system that collects COVID-19 related hospitalization data from 14 different states. https://www.cdc.gov/coronavirus/2019-ncov/covid-data/covid-net/purpose-methods.html

**Table 2:** Presenting vitals and studies of patients hospitalized with COVID-19, total and stratified by presentation

Presentation variable^a^	All hospitalized *n* = 490	Self-presenting *n* = 321	Team-referred *n* = 169	*p*-value
Peak temperature in ER				
°C – median (IQR)	37.2 (36.8–37.9)	37.2 (36.8–38.0)	37.2 (36.9–37.7)	.34
≥ 38 °C – no. (%)	112 (22.9)	84 (26.2)	28 (16.6)	.02
Systolic blood pressure	*n* = 488	*n* = 319	*n* = 169	
mmHg – median (IQR)	133 (119–146)	134 (120–146)	131 (117–145)	.17
Heart rate (BPM) - median (IQR)	96 (86–108)	97 (86–110)	96 (85–103)	**.05**
> 100 BPM – no. (%)	193 (39.4)	141 (43.9)	52 (30.8)	.005
Respiratory rate - median (IQR)	22 (20–26)	22 (20–26)	22 (19–26)	.43
> 20 BPM –no. (%)	276 (56.3)	181 (56.4)	95 (56.2)	.97
Oxygen saturation	*n* = 487	*n* = 318	*n* = 169	
Median percentage (IQR)	91 (87–94)	91 (88–94)	89 (87–93)	.002
Hypoxia (any) – no./n (%)	391/489 (80.0)	236/320 (73.8)	155/169 (91.7)	< .001
None documented – no./n (%)	98 (20.0)	84/320 (26.3)	14/169 (8.3)	< .001
Ambulatory only – no./n (%)	71/489 (14.5)	44/320 (13.8)	27/169 (16.0)	
Resting – no./n (%)	320/489 (65.4)	192/320 (60.0)	128/169 (75.7)	
Chest xray – no./n (%)				
Pneumonia – no./n (%)	380/473 (80.3)	236/305 (77.4)	144/168 (85.7)	.03
Unilateral – no./n (%)	58/380 (15.3)	40/236 (17.0)	18/144 (12.5)	.24
Bilateral – no./n (%)	322/380 (84.7)	196/236 (83.1)	126/144 (87.5)	
Positive blood culture – no./n (%)	28/324 (8.6)	21/207 (10.1)	7/116 (6.0)	.21
GFR (mL/min)	*n* = 478	*n* = 311	*n* = 167	
Median (IQR)	100 (80–115)	103 (84–116)	94 (65–110)	.002
GFR < 60 – no./n (%)	67/485 (13.8)	34/316 (10.8)	33/169 (19.5)	.008
ESRD on dialysis – no./n (%)	18/485 (3.7)	14/316 (4.4)	4/169 (2.4)	
ALT (U/L)	*n* = 486	*n* = 317	*n* = 169	
Median (IQR)	39 (27–58)	41 (27–62)	37 (26–48)	.04
Abnormal ^b^ – no./n (%)	109/486 (22.4)	84/317 (26.5)	25/169 (14.8)	.003
AST (U/L)	*n* = 486	*n* = 317	*n* = 169	
Median (IQR)	52 (40–83)	54 (41–90)	48 (38–74)	.029
Abnormal ^b^ – no./n (%)	315/486 (64.8)	211/317 (66.6)	104/169 (61.5)	.27
Bilirubin (mg/dL)	*n* = 486	*n* = 317	*n* = 169	
Median (IQR)	0.7 (0.5–1.0)	0.7 (0.5–1.1)	0.7 (0.5–0.9)	.60
> 1.3 – no./n (%)	63/486 (13.0)	50/317 (15.8)	13/169 (7.7)	.01
Albumin (g/dL)	*n* = 486	*n* = 317	*n* = 169	
Median (IQR)	4.0 (3.7–4.3)	4.0 (3.7–4.3)	3.9 (3.6–4.2)	.05
WBC count (K/cmm)	*n* = 486	*n* = 317	*n* = 169	
Median (IQR)	6.8 (5.4–9.3)	6.8 (5.3–9.2)	6.7 (5.4–9.3)	.96
< 4.8 – no./n (%)	84/486 (17.3)	53/317 (16.7)	31/169 (18.3)	.65
> 10.8 – no./n (%)	83/486 (17.1)	57/317 (18.0)	26/169 (15.4)	.47
Abnormal (any direction) – no./n (%)	167/486 (34.4)	110/317 (34.7)	57/169 (33.7)	.83
Lymphocyte percent (%)	*n* = 486	*n* = 317	*n* = 169	
Median (IQR)	17.0 (11.4–24.6)	17.00 (11.3–24.4)	16.5 (11.5–25.4)	.81
< 20.5 – no./n (%)	309/486 (63.6)	203/317 (64.0)	106/169 (62.7)	.77
Lymphocyte count (K/cmm)	*n* = 486	*n* = 317	*n* = 169	
Median (IQR)	1149 (841–1527)	1154 (849–1539)	1131 (867–1473)	.96
< 1000 – no./n (%)	183/486 (37.7)	118/317 (37.2)	65/169 (38.5)	.79
Platelet count (K/cmm)	*n* = 485	*n* = 316	*n* = 169	
Median (IQR)	227 (174–285)	227 (171.5–285)	227 (175–286)	.58
< 150 – no./n (%)	77/485 (15.88)	50/316 (15.82)	27/169 (15.98)	.97
Di-dimer (mg/L)	*n* = 438	*n* = 275	*n* = 163	
Median (IQR)	0.78 (0.50–1.25)	0.79 (0.50–1.38)	0.77 (0.50–1.12)	.39
> 0.50 (none < 0.17) – no./n (%)	326/439 (74.26)	205/276 (74.28)	121/163 (74.23)	.99
C-reactive protein (mg/L)	*n* = 445	*n* = 284	*n* = 161	
Median (IQR)	69 (35–162)	66 (32–154)	71 (41–167)	.26
> 50 – no./n (%)	287/461 (62.3)	178/295 (60.3)	109/166 (65.7)	.26
> 100 – no./n (%)	157/461 (34.1)	96/295 (32.5)	61/166 (36.8)	.36
Lactate dehydrogenase (U/L)	*n* = 420	*n* = 263	*n* = 157	
Median (IQR)	723.5 (570–919)	719 (571–920)	728 (563–914)	.95
> 245 – no./n (%)	414/424 (97.6)	258/265 (97.4)	156/159 (98.1)	.62
Creatine phosphokinase (U/L)	*n* = 408	*n* = 262	*n* = 146	
Median (IQR)	84 (48–156.5)	88 (49–185)	79 (47–134)	.05
> 170 – no./n (%)	91/408 (22.3)	71/262 (27.1)	20/146 (13.7)	.002
Troponin (ng/mL)	*n* = 396	*n* = 251	*n* = 145	
> 0.012 – no./n (%)	64/396 (16.2)	42/251 (16.7)	22/145 (15.2)	.68
> 0.040 – no./n (%)	21/396 (5.3)	14/251 (5.6)	7/145 (4.8)	.75
Median of positive (IQR)	0.07 (0.06–0.14)	0.08 (0.05–0.17)	0.07 (0.06–0.13)	.80
Brain natriuretic peptide - (ng/L)	*n* = 90	*n* = 52	*n* = 38	
Median (IQR)	123 (36–1190)	82 (34–412)	258 (54–1283)	.08
> 300 – no./n (%)	33/90 (36.7)	15/52 (28.9)	18/38 (47.4)	.07

*ALT* alanine transaminase, *AST* aspartate transaminase, *BPM* beats per minute, *C* Celsius, *ER* emergency room, *GFR* glomerular filtration rate, *IQR* interquartile range, *WBC* white blood cell

^a^N is listed for each test, as labs were not ordered for all patients. This number includes all numeric lab results included in medians and IQRs; however, non-numeric results (positive/negative or greater/less than threshold) are included in percentages

^b^Abnormal range for ALT and AST are gender specific: For ALT Male > 72 or female > 52. For AST Male > 59 or female > 36

These observations are notable for several reasons. First, contemporary experience suggested that case burdens correlated with mortality rates [25, 26]. The WRSU service community experienced one the highest incidence rates nationally during this time period, with a peak incidence of 430 cases per 100,000 and a peak 7-day average incidence of 240 cases per 100,000 [27, 28]. Second, compared to previous studies, this cohort displayed a high rate of comorbidities associated with severe COVID-19, including an increased prevalence of persons with BMI > 30 (Table 5).

The lower median age of hospitalized persons in our cohort does not sufficiently explain the observed lower mortality rates. When comparing our cohort’s hospital mortality rates to persons in New York City (Supplementary Table 5), the WRSU rates were similar for persons < 50 years, yet markedly lower for persons 50 years of age and older [22]. There 27 (9.3%) deaths among the 290 persons ≥50 years of age, and 24 (14%) deaths among the 166 persons ≥60 years of age hospitalized for COVID-19 related illness, both proportions that are significantly lower than stratified percentages reported by the CDC over the same time period [24]. In summary, when compared to the general population, our service community had an elevated per-capita disease burden, comparable hospitalization rates, yet lower overall case fatality rates and in-hospital mortality. We believe the primary driver for this observed improvement in mortality was the outsized reduction in deaths among the oldest hospitalized persons.

The observational cohort studies in Table 5 were chosen as comparators partly based on the similar study design and limitations. There are obvious challenges with such comparisons. As such, we compared WRSU demographics and outcomes with the Adaptive Covid-19 Treatment Trials, ACTT-1 and ACTT-2 (Supplementary Table 6) [19, 29]. These trials were significantly more heterogenous demographically and geographically, creating more representative generalizable sample with which to compare the WRSU experience. Compared to ACTT-1, WRSU patients were younger, more likely to be obese, and more likely to have one or ≥ 2 co-morbidities [ 19]. Compared to ACTT-2, WRSU patients were comparable in age and obesity rates, but more likely to have one or ≥ 2 co-morbidities comorbidities [ 29]. What is most relevant is that the WRSU in-hospital mortality (7%) was significantly lower than not only the whole cohort of ACTT-1 (13%) but lower than the treatment arm (11%) in which 100% of the subjects received the anti-viral remdesivir. And while the in-hospital moralities were comparable between WRSU (7%) and ACTT-2 (6%), it is worth noting that 100% of both arms received some therapy, either remdesivir (CFR 8%) or remdesivir plus baricitinib (CFR 5%). Lastly, it is important to point out that < 1% of subjects ACTT-1/− 2 were American Indian or Alaska Native, where as 100% of the WRSU patients are American Indian. Like many displaced, rural, indigenous populations, American Indians in the southwest face a myriad of challenges to accessing health care as well as numerous health disparities, the discussion of which is beyond the scope of this manuscript. Nonetheless, the differences do not suggest that the WRSU experience achieved notable outcomes because access was better or because the local population was healthier.

### Patient characteristics

The demographic characteristics of persons hospitalized with COVID-19 at WRSU differ from previous studies. Most notably, the median age of hospitalized persons in our cohort (54 years) was younger by nearly 10 years compared to similar studies [6, 10, 20–23]. This may be attributable to higher rates of underlying conditions in American Indian communities acquired at a younger age [30], and further supported by the finding that despite the younger age range, over 90% of the WRSU cohort had at least one comorbidity (Table 1, Table 5). This was particularly apparent with obesity, as 60% of persons at WRSU had a BMI over 30 compared to a maximum of 42% in other studies (Table 5). Again, it is important to note that the lower median age does not necessarily translate into reduced risk, particularly given the evidence that after age, BMI is one of the strongest predictors of severe COVID-19 [31, 32].

We observed high rates of substance use among younger persons that required hospitalization for severe COVID-19, suggesting this may be an additional risk factor for severe COVID-19 for medical or psychosocial reasons. Methamphetamine abuse, particularly inhaled, leads to baseline lung injury and modulates immune function, and methamphetamine use has been associated with increased risk of pulmonary diseases [33, 34]. Alcohol use also impacts the immune system, making the body more susceptible to infectious disease [35–37]. Although substance use rates vary widely between communities, alcohol and drug-related mortality rates are higher in American Indian populations [38]. The elevated prevalence of alcohol and substance in our cohort could be contributing to the lower median age of severe disease. Previous research has not provided clear answers on the role of substance abuse in COVID-19 and this gap could lead to an under-appreciation of the impact substance and alcohol abuse has in younger COVID-19 positive persons.

As observed in previous studies, we found high rates of hypertension, diabetes mellitus, and obesity among hospitalized persons. Our findings that older age, male sex, and higher BMI are associated with COVID-19 mortality in hospitalized persons support previous findings.

### Potential impact of referral system

It is difficult to compare persons who were referred by the tracing team to persons who self-presented, as the tracing team specifically focused on following persons believed to be at the highest risk for decompensation. We cannot make causal claims from our observations for various reasons. While referred persons actually had higher overall and in-hospital mortality, we do not suspect close follow-up and early intervention led to this finding. Rather, we believe the mortality rates would have been substantially higher for this cohort had there not been a high-risk referral program in place. This is supported by our previously published work on the effective and efficient Case Investigation and Contact Tracing program at the WRSU and the mortality reductions directly observed through our high-risk outreach activities [15, 16, 39].

Additionally, it is likely that actively following persons allowed for more accurate and complete data on symptoms, which could skew symptom onset dates earlier than for persons who self-reported symptoms based on their memories. Despite these analytical challenges, it is notable that the median time from symptom onset to hospitalization at WRSU was 4 days (IQR 2–7), which is much lower than reported elsewhere (7 days, IQR 3–9) [23, 40]. Earlier intervention could prevent persons from becoming progressively hypoxic at home where the absence of supplemental oxygen leads to cascading organ injury. Whereas in the hospital, in addition to oxygen, early referral allows to optimal administration of the anti-viral remdesivir that has shown to be more effective earlier in the course of disease [19].

### Limitations

We recognize several limitations. First, this data is site-specific and is restricted to persons who were evaluated at the Whiteriver Service Unit. While it helps fill a gap regarding COVID-19 in American Indian populations, it is not necessarily generalizable to other Indigenous communities, nor the general population. In addition, this study suffers from the limitations inherent to retrospective observational studies. We cannot draw conclusions about causation, and some records had missing data. It is also difficult to draw any concrete conclusions regarding the high-risk outreach program, given that this program was not randomized in any way. Despite these limitations, we feel that this data is an important addition to the current information on COVID-19 in American Indian populations.

## Conclusions

We observed one of the lowest overall and in-hospital case fatality rates during the early months of the COVID-19 pandemic in a rural, American Indian population that concomitantly experienced one of the highest incidences of COVID-19 in 2020. We believe integrated contact tracing and the high-risk outreach programs paired with an early referral system mitigated a significant amount of morbidity and mortality secondary to COVID-19. This shows the value of an integrated community-based response. While our findings may not be generalizable, the system remains a potentially good model for communities served by centralized healthcare systems or in rural settings.

### Supplementary Information

**Supplementary material 1.**

## Data Availability

Data collected on Tribal lands are owned by the participating Tribal Nations. Data can be made available upon request to the corresponding author (contact Ryan.M.Close@gmail.com) if consistent with the Institutional Review Board-approved protocol and if the disclosure is approved by the participating Tribes.

## Abbreviations

AST/ALT: Aspartate transaminase/alanine transaminase
BMI: Body mass index
BNP: B-type natriuretic peptide
COVID-19: Novel coronavirus disease 2019
CPK: Creatine phosphokinase
CRP: C-reactive protein
GFR: Glomerular filtration rate
ICU: Intensive care unit
IHS: Indian Health Service
IQR: Interquartile range
SD: Standard deviation
WRSU: Whiteriver service unit
