# Supplementary material for: Characteristics and outcomes of a hospitalized cohort with reduced mortality from COVID-19, White Mountain apache tribal lands, April 1 – July 31, 2020
Source: BMC Public Health. 2024 Mar 1;24:648. doi: 10.1186/s12889-024-18098-5 (PMC10905852; doi:10.1186/s12889-024-18098-5)
Supplement: Supplementary file 1 — Supplementary material 1. [file 12889_2024_18098_MOESM1_ESM.docx]

**Supplementary Material**

**Supplementary Table 1.** Symptoms on presentation of patients hospitalized with COVID-19, total and stratified by presentation.

| Symptom | All hospitalized  n = 490 | Self-presenting  n = 294 | Team-referred  n = 162 | p-value |
| --- | --- | --- | --- | --- |
| Asymptomatic – no./n (%) | 32/488 (6.6) | 25/319 (7.8) | 7/169 (4.1) | .12 |
| Symptomatic – no./n (%) | 456/488 (93.4) | 294/319 (92.2) | 162/169 (95.9) |  |
| Symptom to hospitalization interval - Days -- median (IQR) | 4 (2-7) | 4 (2-7) | 5 (2-8) | .003 |
| Subjective Fever – no. (%) | 264 (57.9) | 172 (58.5) | 92 (56.8) | .72 |
| Chills – no. (%) | 163 (35.8) | 99 (33.7) | 64 (39.5) | .21 |
| Aches – no. (%) | 222 (48.7) | 130 (44.2) | 92 (56.8) | .01 |
| Fatigue – no. (%) | 124 (27.2) | 73 (24.8) | 51 (31.5) | .13 |
| Rhinorrhea – no. (%) | 42 (9.2) | 23 (7.8) | 19 (11.7) | .17 |
| Sore Throat – no. (%) | 46 (10.1) | 27 (9.2) | 19 (11.7) | .39 |
| Cough – no. (%) | 347 (76.1) | 214 (72.8) | 133 (82.1) | .03 |
| Shortness of Breath – no. (%) | 336 (73.7) | 203 (69.1) | 133 (82.1) | .002 |
| Nausea – no. (%) | 119 (26.1) | 81 (27.6) | 38 (23.5) | .34 |
| Vomiting – no. (%) | 87 (19.1) | 66 (22.5) | 21 (13.0) | .01 |
| Diarrhea – no. (%) | 110 (24.1) | 65 (22.1) | 45 (27.8) | .18 |
| Abdominal Pain – no. (%) | 53 (11.6) | 40 (13.6) | 13 (8.0) | .08 |
| Headache – no. (%) | 127 (27.9) | 72 (24.5) | 55 (34.0) | .03 |
| Loss of Taste – no. (%) | 44 (9.7) | 26 (8.8) | 18 (11.1) | .43 |
| Loss of Smell – no. (%) | 34 (7.5) | 20 (6.8) | 14 (8.6) | .47 |
| Chest Pain – no. (%) | 94 (20.6) | 54 (18.4) | 40 (24.7) | .11 |
| Altered Mental Status – no. (%) | 16 (3.5) | 12 (4.1) | 4 (2.5) | .43 |

**Supplementary Table 2.** Additional outcomes of patients hospitalized with COVID-19, total and stratified by presentation.

| Characteristic | All hospitalized  n = 490 | Self-presenting  n = 321 | Team-referred  n = 169 | p-value |
| --- | --- | --- | --- | --- |
| Sepsis | 68/457 (14.88) | 44/300 (14.67) | 24/157 (15.29) | .86 |
| Extracorporeal membrane oxygenation | 2/456 (0.44) | 0/299 (0.00) | 2/157 (1.27) | .12 |
| Acute respiratory distress syndrome | 29/456 (6.36) | 17/299 (5.69) | 12/157 (7.64) | .42 |
| Arrhythmia | 16/456 (3.51) | 8/299 (2.68) | 8/157 (5.10) | .18 |
| Acute coronary syndrome | 3/456 (0.66) | 3/299 (1.00) | 0/157 (0.00) | .55 |
| Cardiomyopathy | 6/456 (1.32) | 5/299 (1.67) | 1/157 (0.64) | .67 |
| Congestive heart failure | 4/456 (0.88) | 2/299 (0.67) | 2/157 (1.27) | .61 |
| Vasopressor requirement | 42/456 (9.21) | 25/299 (8.36) | 17/157 (10.83) | .39 |
| Venous thromboembolism | 6/456 (1.32) | 4/299 (1.34) | 2/157 (1.27) | 1.0 |
| Cerebrovascular accident | 6/456 (1.32) | 4/299 (1.34) | 2/157 (1.27) | 1.0 |
| Renal replacement therapy | 12/456 (2.63) | 7/299 (2.34) | 5/157 (3.18) | .59 |
| Acute kidney injury | 53/459 (11.55) | 26/300 (8.67) | 27/159 (16.98) | .008 |

**Supplementary Table 3.** Multiple logistic regression for association of comorbidities with COVID-19 death within 28 days.

| Independent variable | Variable type | Coding | Coefficient | Standard error | t-value | p-value |
| --- | --- | --- | --- | --- | --- | --- |
| Age | Continuous | Years | .081 | .015 | 5.46 | < .001 |
| BMI | Continuous | BMI value | .068 | .021 | 3.16 | .002 |
| Male Sex | Dichotomous | 0-1, 1=male | .759 | .359 | 2.11 | .04 |
| Constant | N/A | N/A | -9.91 | 1.505 | -6.58 | < .001 |
| Response variable: COVID-19 death within 30 days (dichotomous, 0=no death, 1=COVID-19 death). Excludes non-COVID-19 related deaths.  Overall Model: Pseudo r-squared = 0.16, chi-square = 43.4, p-value <.001 | | | | | | |

**Supplementary Table 4.** Multiple logistic regression for association of comorbidities with peak inpatient oxygen delivery mode.

| Independent variable | Variable type | Coding | Coefficient | Standard error | t-value | p-value |
| --- | --- | --- | --- | --- | --- | --- |
| Age | Continuous | Years | .081 | .015 | 5.46 | < .001 |
| BMI | Continuous | BMI value | .068 | .021 | 3.16 | .002 |
| Male Sex | Dichotomous | 0-1, 1=male | .759 | .359 | 2.11 | .04 |
| Constant | N/A | N/A | -9.91 | 1.505 | -6.58 | < .001 |
| Response variable: Peak inpatient oxygen delivery mode (ordinal, 0-7, None < NC < NRB < HFNC < CPAP < BiPAP < Mechanical ventilation)  Overall Model: Pseudo r-squared = 0.12, chi-square = 50.1, p-value < .001 | | | | | | |

**Supplementary table 5.** COVID-19 hospital mortality by age group at WRSU and in New York City.

| Outcome | WRSU | NYC^1^ | p-value |
| --- | --- | --- | --- |
| Overall hospital mortality – no./n (%) | 35/490 (7) | 553/2634 (21) | <0.001 |
| Age < 50 – no./n (%) | 8/200 (4) | 34/694 (5) | .60 |
| Age 50-59 – no./n (%) | 3/124 (2) | 53/515 (10) | .005 |
| Age 60-69 – no./n (%) | 7/86 (8) | 84/533 (16) | .06 |
| Age 70-79 – no./n (%) | 8/57 (14) | 145/451 (32) | .02 |
| Age ≥ 80 – no./n (%) | 9/23 (39) | 237/441 (54) | .17 |
| ^1^Richardson S, Hirsch JS, Narasimhan M, et al. Presenting Characteristics, Comorbidities, and Outcomes Among 5700 Patients Hospitalized With COVID-19 in the New York City Area. *JAMA*. 2020;323(20):2052-2059. doi:10.1001/jama.2020.6775 | | | |

**Supplementary table 6.** Comparing the Whiteriver Service Unit cohort of patients hospitalized with COVID-19 to ACTT-1 and ACTT-2

| **Characteristics** | **Whiteriver Service Unit** | **Beigel et al. – ACTT-1**^1^ | | | **Kalil et al. – ACTT-2**^2^ | | |
| --- | --- | --- | --- | --- | --- | --- | --- |
| **Location** | Whiteriver, AZ, USA | All | Remdesivir | Placebo | All | Remdesivir + Baricitinib | Remdesivir + Placebo |
| **n** | 490 | 1062 | 541 | 521 | 1033 | 515 | 518 |
| **Demographics** | n=490 | n=1062 | n=541 | n=521 | n=1033 | n=515 | n=518 |
| Age -- median (IQR) | 54 (41-64) | 59 (15)* | 58 (15)* | 59 (15)* | 55 (16)* | 55 (15)* | 56 (16)* |
| Female sex -- no. (%) | 269 (55) | 378 (36 | 189 (35) | 189 (36) | 381 (37) | 196 (38) | 185 (36) |
| Obesity -- no./n (%) | 290/486 (60) | 476/1049 (45) | 242/531 (46) | 234/518 (45) | 295 (58) | 272 (53) | 567 (56) |
| **No. Comorbidities** |  |  |  |  |  |  |  |
| At least one -- no. (%) | 451 (92) | 854/1048 (82) | 434/531 (82) | 420/517 (81) | 839/994 (84) | 432/496 (87) | 407/498 (82) |
| More than one -- no. (%) | 351 (72) | 579/1048 (55) | 296/531 (56) | 283/517 (55) | 569/994 (57) | 284/496 (57) | 285/498 (57) |
| **Outcomes** |  |  |  |  |  |  |  |
| ICU -- no./n (%) | 95/463 (21) | -- | -- | -- | -- | -- | -- |
| Intubation -- no./n (%) | 51/464 (11) | 285 (27) | 131 (24) | 154 (30) | 116/922 (13) | 46/461 (10) | 70/461 (15) |
| Death -- no./n (%) | 35/490 (7) | 136/1062 (13)** | 59 (11) | 77 (15) | 61 (6)** | 24 (5) | 37 (8) |
| *ACTT-1 and ACTT-2 reported age as mean (SD)  ** This table reports crude case fatality rates for all subjects in ACTT-1 and ACCT-2, along with the Kaplan-Meier reported mortality by study arm.  ^1^ Beigel, J. H., et al. (2020). "Remdesivir for the Treatment of Covid-19 — Final Report." New England Journal of Medicine **383**(19): 1813-1826.  ^2^ Kalil, A. C., et al. (2020). "Baricitinib plus Remdesivir for Hospitalized Adults with Covid-19." New England Journal of Medicine **384**(9): 795-807 | | | | | | | |
